# Assessment of Relative Utility of Underlying vs Contributory Causes of Death

**DOI:** 10.1001/jamanetworkopen.2019.8024

**Published:** 2019-07-31

**Authors:** G. David Batty, Catharine R. Gale, Mika Kivimäki, Steven Bell

**Affiliations:** 1 School of Biological and Population Health Sciences, Oregon State University, Corvallis; 2 Department of Epidemiology and Public Health, University College London, London, United Kingdom; 3 Medical Research Council Lifecourse Epidemiology Unit, University of Southampton, Southampton, United Kingdom; 4 Centre for Cognitive Ageing and Cognitive Epidemiology, University of Edinburgh, Edinburgh, United Kingdom; 5 Cardiovascular Epidemiology Unit, Department of Public Health and Primary Care, University of Cambridge, Cambridge, United Kingdom; 6 The National Institute for Health Research Blood and Transplant Unit in Donor Health and Genomics, University of Cambridge, Cambridge, United Kingdom

## Abstract

**Importance:**

In etiological research, investigators using death certificate data have traditionally extracted underlying cause of mortality alone. With multimorbidity being increasingly common, more than one condition is often compatible with the manner of death. Using contributory cause plus underlying cause would also have some analytical advantages, but their combined utility is largely untested.

**Objective:**

To compare the relative utility of cause of death data extracted from the underlying cause field vs any location on the death certificate (underlying and contributing combined).

**Design, Setting, and Participants:**

This study compares the association of 3 known risk factors (cigarette smoking, low educational attainment, and hypertension) with health outcomes based on where cause of death data appears on the death certificate in 2 prospective cohort study collaborations (UK Biobank [N = 502 655] and the Health Survey for England [15 studies] and the Scottish Health Surveys [3 studies] [HSE-SHS; N = 193 873]). Data were collected in UK Biobank from March 2006 to October 2010 and in HSE-SHS from January 1994 to December 2008. Data analysis began in June 2018 and concluded in June 2019.

**Main Outcomes and Measures:**

Death from cardiovascular disease, cancer, dementia, and injury. For each risk factor–mortality end point combination, a ratio of hazard ratios (RHR) was computed by dividing the effect estimate for the underlying cause by the effect estimate for any mention.

**Results:**

In UK Biobank, there were 14 421 deaths (2.9%) during a mean (SD) of 6.99 (1.03) years of follow up; in HSE-SHS, there were 21 314 deaths (11.0%) during a mean (SD) of 9.61 (4.44) years of mortality surveillance. Established associations of risk factors with death outcomes were essentially the same irrespective of placement of cause on the death certificate. Results from each study were mutually supportive. For having ever smoked cigarettes (vs never having smoked) in the UK Biobank, the RHR for cardiovascular disease was 0.98 (95% CI, 0.87-1.10; *P *value for difference = .69); for cancer, the RHR was 0.99 (95% CI, 0.93-1.05; *P *value for difference = .69). In the HSE-SHS, the RHR for cardiovascular disease was 0.94 (95% CI, 0.87-1.01; *P *value for difference = .09); for cancer, it was 1.01 (95% CI, 0.94-1.10; *P* value for difference = .75).

**Conclusions and Relevance:**

Risk factor–end point associations were not sensitive to the placement of data on the death certificate. This has implications for the examination of the association of risk factors with causes of death where there may be too few events to compute reliable effect estimates based on the underlying field alone.

## Key Points

**Question:**

What is the relative utility for etiological research of using underlying cause of mortality from death certificates compared with using any field?

**Findings:**

In 2 large cohort studies with 696 528 participants combined, associations of known risk factors with an array of death outcomes were essentially the same, irrespective of the location of the death data on the certificate.

**Meaning:**

Our findings have implications for risk factor–end point computations in studies of rarer outcomes and those with smaller sample sizes.

## Teaser

This cohort study, using data from the UK Biobank and health surveys in England and Scotland, compares the relative utility of cause of death data extracted from the underlying cause field vs any location on the death certificate.

## Introduction

Death records have long been collected for the purposes of monitoring the health of populations,^1,2^ quantifying disease prognosis,^3,4^ and evaluating the impact of primary^5^ and secondary interventions.^6^ To examine the influence of environmental and genetic characteristics on disease and injury events, mortality records have also been extensively deployed in ecological,^7^ case-control,^8^ experimental,^9^ and, most frequently, prospective cohort studies.^10,11,12^ The use of death records as a proxy for a health end point of interest is particularly important in contexts where linkages to other electronic health registries, such as hospital data or cancer records, are not viable, and clinical examination of study members is financially or logistically prohibitive. Linkage to death registers has the further advantage of having no additional burden on study participants. Scientific endeavors in which death data have been central to the understanding of disease etiology include the Framingham studies, where hypertension was first shown to be a risk factor for heart disease^13^ and stroke^14^; the original Whitehall study,^15^ where it was demonstrated that elevated blood glucose within the normal range was related to vascular events; and the British Doctors Study^16^ where, perhaps most famously, smoking was first prospectively linked to lung cancer. There are numerous other examples.^17^

To accord with the World Health Organization guidelines,^18^ death certificates are formulated in 2 parts. For the purposes of epidemiological research, the underlying (ie, immediate or direct) cause of death is almost exclusively extracted. Other diseases or injuries that contributed to the death but were not directly implicated appear in another section of the certificate. However, in practice, this contributory information is very rarely used.^19^ With multimorbidity being common in an era of effective treatments, more than one condition can be compatible with the manner of death.^20^ Analyses that use only the underlying cause of death may therefore omit valuable information that is readily available.

In estimating burden of disease, reliance on underlying cause compared with incorporating contributory causes appears to lead to underestimates of the importance of several leading causes of death.^21^ However, the impact for etiological research is largely unknown. In the only study of which we are aware,^22^ investigators found the same predictive capacity for classic risk factors in analyses featuring cardiovascular disease deaths irrespective of placement on the death certificate. No such comparison was made for other important causes of death. Using data from the contributory field of death may have the analytical advantage of facilitating investigation of the determinants of rarer causes of death (eg, intentional injury^23^ and dementia^24^) where, particularly in smaller cohort studies, a reliance on underlying cause alone may result in too few events to facilitate statistical computations. The value to investigators of larger studies of commonly occurring conditions might be enhanced statistical precision.

Using data from 2 large cohort studies, we examined associations of 3 known risk factors with major causes of death, including cancer, cardiovascular disease, injury, and dementia. To provide findings of interest to a range of disciplines, we used physiological (hypertension^25^), psychosocial (educational attainment^26^), and behavioral (cigarette smoking^27,28^) risk factors. Our aim was to examine if these risk factors had the same magnitude of association with cause-specific mortality when end point data were extracted from the underlying field alone vs the underlying and contributory fields combined (ie, any mention).

## Methods

### Included Cohort Studies

We used data from UK Biobank,^29^ a prospective cohort study, and a pooling of 18 identical cohort studies from the Health Survey for England (15 studies) and the Scottish Health Surveys (3 studies) (HSE-SHS).^30,31,32^ These studies were selected because they offer similar, standard processes for data collection. Participants in both studies gave full informed consent. In the UK Biobank, ethical approval was received from the North West Multi-center Research Ethics Committee, and the research was carried out in accordance with the Declaration of Helsinki.^33^ In HSE-SHS, ethical approval for data collection was granted by the London research ethics council or the local research ethics councils. This study analyzed existing anonymized data, and therefore, no further ethical approval was required. This report follows the Strengthening the Reporting of Observational Studies in Epidemiology (STROBE) reporting guideline.

### Baseline Data Collection

The sampling and protocols of these studies have been well described.^29,30,31,32^ In brief, baseline data collection in the UK Biobank took place between March 2006 and October 2010 in research assessment centers across the United Kingdom, with data collected on 502 655 people aged 40 to 69 years (response proportion, 6%). Between January 1994 and December 2008, a total of 193 842 people aged 16 to 102 years (response proportion, 64%-78%) participated in HSE-SHS, with data collection taking place exclusively in the home (Table).

**Table.** Characteristics of Study Members in UK Biobank and HSE-SHS

Characteristic	No. (%)	
	UK Biobank	HSE-SHS
Participants recruited, No.^a^	502 655	193 873
Women	273 472 (54.4)	106 469 (54.9)
Age at baseline, mean (SD) [range], y	56.5 (8.1) [38-73]	46.8 (18.5) [16-102]
Ever smoked	225 896 (45.2)	98 581 (51.1)
No university education	331 298 (67.3)	147 353 (76.0)
Hypertension	282 637 (57.2)	43 428 (36.9)
Duration of mortality surveillance, mean (SD), y	6.99 (1.03)	9.61 (4.44)
Death from any cause	14 421 (2.9)	21 314 (11.0)

Abbreviation: HSE-SHS, Health Survey for England and the Scottish Health Surveys.

^a^ Number of study members and events are higher in this Table than in the survival analyses, which have missing data.

Responses to history of cigarette smoking habits (ie, ever smoker vs never) and highest attained educational qualification (ie, no university degree vs ≥university undergraduate degree) were collapsed into binary categories for the purposes of presentation brevity. In UK Biobank, systolic and diastolic blood pressure measurements were taken twice while the participant was seated using the Omron HEM-7015IT digital blood pressure monitor (Omron Healthcare).^20^ Blood pressure in the present analyses was based on the average of the 2 measurements. In HSE-SHS, blood pressure was measured using the Dinamap 8100 automated device (GE Critikon).^34^ Following a 5-minute seated rest, 3 readings of systolic and diastolic blood pressure were taken from the right arm at 1-minute intervals. Blood pressure used in the present analysis was based on the mean of the second and third measurement. We defined hypertension according to existing guidelines as systolic/diastolic blood pressure of at least 140/90 mm Hg and/or use of antihypertensive medication.^35^

### Ascertainment of Cause-Specific Mortality

Study participants were flagged using the procedures of the UK National Health Service (NHS) Central Registry. Underlying and contributing deaths were classified according to the *International Statistical Classification of Diseases and Related Health Problems, Tenth Revision *(*ICD-10*)^18^ as follows: attributable to (1) all cancers combined (codes C00-C97), (2) lung cancer (C34), (3) cardiovascular disease (I20-5, I50, I60-70, I73, and I74), (4) coronary heart disease (I20-5), (5) cerebrovascular disease (I60-9), (6) external causes (V01-Y99), and (7) dementia (F00-F02, F03, F05, F10, G30, G31, I67 and A81).^24^ Where necessary, corresponding codes from earlier revisions of the *ICD* were used. The any mention category was a combination of the underlying and contributory cause of death fields.

### Statistical Analysis

Hazard ratios (HRs) and accompanying 95% CIs were computed using Cox regression models^36^ and adjusted for age and sex. In these survival analyses, we censored individuals according to the date of death or the end of follow-up (February 22, 2016, for UK Biobank, February 14, 2011, for HSE, and December 31, 2009, for SHS), whichever came first. For a summary measure of the difference between the HRs based on the 2 approaches to classifying mortality, we computed a ratio of the HRs (RHR) as has been used elsewhere.^37,38^ We also computed the *P* value for difference between underlying vs any mention HRs for the 2 studies. To do so, we used the Fisher *z* score measure to test for equality of HRs, with *z *calculated as the difference between the logarithms of the HRs (β1, β2) divided by the square root of the sum of the square of their variances (SE1^2^, SE2^2^), where *z* follows a normal distribution: *z* = (β1 − β2)/√(SE1^2^ + SE2^2^).^39^ No prespecified level of statistical significance was set, and all tests were 2-tailed. Analyses were conducted using Stata version 15 (Stata Corp) and took place from June 2018 to June 2019.

## Results

In the Table, we show the characteristics of study members in UK Biobank and HSE-SHS at baseline and their mortality experience. The proportion of women in the baseline sample was similar in UK Biobank (273 472 [54.4%]) and HSE-SHS (106 469 [54.9%]). The prevalence of people who reported ever having smoked and those without a university education was somewhat higher in HSE-SHS than UK Biobank (smoking: 98 581 [51.1%] vs 225 896 [45.2%]; no university degree: 147 353 [76.0%] vs 331 298 [67.3%]), while hypertension was less common (43 428 [36.9%] vs 282 637 [57.2%]).

**Figure 1.**
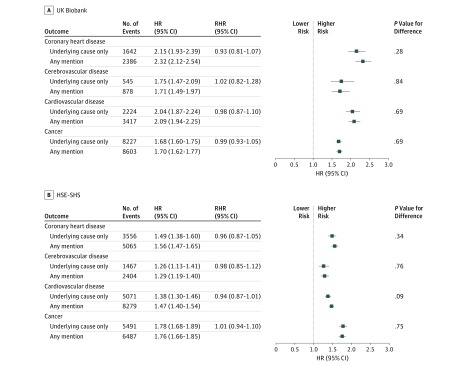
Association of Smoking Status With Cause-Specific Mortality in the UK Biobank (N = 499 701) and the Health Survey for England and Scottish Health Surveys (HSE-SHS; N = 193 037) Shaded squares indicate the hazard ratios (HRs), and error bars denote the 95% CIs for the association of smoking status with the risk of death from a range of diseases. The reference group is never having smoked cigarettes. The ratio of hazard ratios (RHR) summarizes the difference, with underlying cause as the reference group, between the effect estimate for the outcome as ascertained from different locations on the death certificate. The number of study participants and deaths in the sample used in this survival analysis is marginally lower than the full cohort owing to missing data for the exposure of interest.

**Figure 2.**
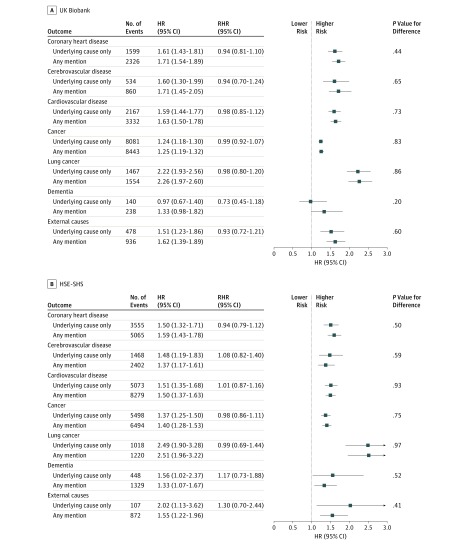
Association of Educational Attainment with Cause-Specific Mortality in the UK Biobank (N = 492 513) and the Health Survey for England and Scottish Health Surveys (HSE-SHS; N = 193 702) Shaded squares indicate the hazard ratios (HRs), and error bars denote the 95% CI for the association of educational attainment with the risk of death from a range of long-term diseases and injury. The reference group is having a university undergraduate degree or higher. The ratio of hazard ratios (RHR) summarizes the difference, with underlying cause as the reference group, between the effect estimate for the outcome as ascertained from different locations on the death certificate. The number of study participants and deaths in the sample used in this survival analysis is marginally lower than the full cohort owing to missing data for the exposure of interest.

**Figure 3.**
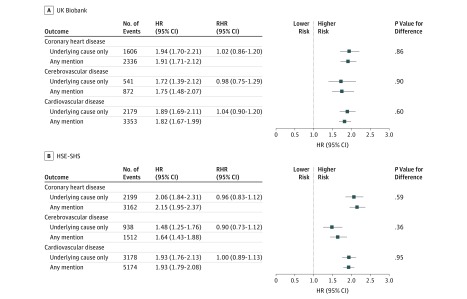
Association of Hypertension Status With Cardiovascular Disease Mortality in the UK Biobank (N = 492 513) and the Health Survey for England and Scottish Health Surveys (HSE-SHS; N = 117 606) Shaded squares indicate the hazard ratios (HRs), and error bars denote the 95% CIs for the association of hypertension status with the risk of death from different presentations of cardiovascular disease. The reference group is not having hypertension. The ratio of hazard ratios (RHR) summarizes the difference, with underlying cause as the reference group, between the effect estimate for the outcome as ascertained from different locations on the death certificate. The number of study participants and deaths in the sample used in this survival analysis is marginally lower than the full cohort owing to missing data for the exposure of interest.

In UK Biobank there were 14 421 deaths among 502 655 people (2.9%) during a mean (SD) follow-up period of 6.99 (1.03) years, while in the pooling of HSE-SHS, a mean (SD) follow-up period of 9.61 (4.44) years of mortality surveillance gave rise to 21 314 deaths among 193 873 individuals (11.0%). In Figure 1, Figure 2, and Figure 3, we show numbers of deaths for each major cause of mortality as retrieved from the underlying cause field as well as in combination with the contributing field (ie, any mention). The number of study participants and deaths in the sample used in the Figures is marginally lower than the full cohort owing to some missing data for the exposures of interest. The number of deaths in the any mention group was necessarily higher for all conditions, a differential that was least pronounced for cancer, which may reflect dissemination of the primary malignancy (ie, underlying cause).

In Figure 1, we show the age-adjusted and sex-adjusted HRs for baseline cigarette smoking status associated with deaths from cardiovascular disease, its different presentations (ie, coronary heart disease and cerebrovascular disease), and all cancers combined. The expected associations were apparent, such that ever having smoked cigarettes was associated with an elevated rate of mortality from all conditions. Within studies, the size of the effect estimates was very similar, irrespective of whether the underlying or any mention field was used, so that the RHRs all hovered around unity (*P* value for difference ≥ .09). For example, in the UK Biobank, the RHR for cardiovascular disease was 0.98 (95% CI, 0.87-1.10; *P *value for difference = .69); for cancer, the RHR was 0.99 (95% CI, 0.93-1.05; *P *value for difference = .69). In the HSE-SHS, the RHR for cardiovascular disease was 0.94 (95% CI, 0.87-1.01; *P *value for difference = .09); for cancer, it was 1.01 (95% CI, 0.94-1.10; *P* value for difference = .75). Similar observations were apparent for lung cancer, which, owing to the strong association with smoking in both UK Biobank (underlying cause: HR, 7.60; 95% CI, 6.50-8.88; any mention: HR, 7.84; 95% CI, 6.72-9.13) and HSE-SHS (underlying cause: HR, 10.38; 95% CI, 8.14-13.23; any mention: HR, 9.04; 95% CI, 7.33-11.15) could not be accommodated in the Figure alongside the expected weaker effect estimates for other outcomes. Owing to the higher numbers of deaths in the any mention group, statistical precision was somewhat higher, as evidenced by the tighter 95% CIs.

In Figure 2 we show HRs for the association of educational attainment with the same mortality outcomes featured in Figure 1, with the addition of dementia and external causes. As anticipated, a more basic education, denoted by the absence of a university degree, was associated with an increased risk of mortality from all featured causes of death. The magnitude of these effect estimates was similar within the studies irrespective of the position of cause on the death certificate; thus, the RHRs ranged from 0.73 (95% CI, 0.45-1.18) for dementia in UK Biobank to 1.30 (95% CI, 0.70-2.44) for external causes in HSE-SHS, with a *P* value for difference > .20.

Last, in Figure 3 we depict the association of hypertension with mortality risk. People with hypertension experienced elevated rates of death from cardiovascular disease compared with their unaffected counterparts. Cross-study HRs were very similar with the RHRs all being close to unity and the *P* values for difference all being nonsignificant at conventional levels. Thus, the RHRs for cardiovascular disease in UK Biobank (1.04; 95% CI, 0.90-1.20; *P* value for difference = .60) and in HSE-SHS (1.00; 95% CI, 0.89-1.13; *P* value for difference = .95) were very similar, as they were for its subtypes of coronary heart disease (UK Biobank: RHR, 1.02; 95% CI, 0.86-1.20; *P* value for difference = .86; HSE-SHS: RHR, 0.96; 95% CI, 0.83-1.12; *P* value for difference = .59), and cerebrovascular disease (UK Biobank: RHR, 0.98; 95% CI, 0.75-1.29; *P* value for difference = .90; HSE-SHS: RHR, 0.90; 95% CI, 0.73-1.12; *P* value for difference = .36).

## Discussion

In this study, known associations of risk factors with an array of health end points were essentially the same irrespective of whether death data were drawn from the underlying cause field on the death certificate or a combination of underlying and contributory categories. These observations were confirmed in independent data sets. The any mention field is, as described, a combination of underlying and contributory fields. For outcomes where there is a small difference in absolute numbers of cases between these groups, such as cancer, the HRs based on analyses of each group will necessarily be nearly identical. More surprising is the similarity in effect estimates where discordance in the number of events is high, that is, for all other outcomes featured herein: cardiovascular disease (and the different presentations it comprises), dementia, and external causes. An implication of our findings is that using the contributory field alongside the underlying cause field may have the advantage of facilitating investigation of risk factors for the occurrence of rarer forms of death where, particularly in smaller cohort studies, there may be too few events to compute effect estimates using the underlying field alone. The value in larger studies, such as those used here, might be marginally improved statistical precision.

As described, we were able to identify only 1 other study that has systematically compared the utility for etiological research of using a combination of the underlying cause and contributory cause fields on death certificates with using the underlying cause filed alone.^22^ Using mortality records from the Western Electric Study, the 5 risk factors examined—age, systolic blood pressure, blood cholesterol, body mass index, and cigarette smoking status—revealed near-identical HRs for the association of these risk factors with cardiovascular disease mortality for the 2 sources of the mortality outcome. There are also examples of investigators who have followed this analytical process but reported their findings qualitatively only. Thus, in our previous work,^40^ psychological distress was associated with an elevated risk of death as drawn from the major *ICD*-*10* chapters, whether extracted from underlying cause on the death certificate or in combination with contributory cause. Similar results were seen when the predictive capacity of alcohol intake and obesity for liver disease was assessed,^41^ when pulmonary function was associated with dementia death,^42^ and when we investigated the association of neuroticism with mortality from various causes.^43^

### Strengths and Limitations

While the present study has strengths—its relative novelty and the comparison of results across large, well-powered studies—there are inevitably some shortcomings. Biomedical data, while available for analyses in HSE-SHS, were not available in UK Biobank at the time of analyses. It has therefore not been possible to compare the association of cholesterol fractions, glycated hemoglobin, and inflammatory markers, all linked etiologically with cardiovascular disease,^44^ with mortality across different placements of cause of death on the death certificate. Based on the present results, we think it is unlikely that these risk indices will yield results very different from the patterns described here. Second, our exposure variables are known to be associated with the major stroke subtypes (ie, ischemic and hemorrhagic); however, on UK death certificates stroke subtype is usually too ill defined to be useful.^45^ Therefore, we were not able to run such analyses. Third, while UK Biobank is undoubtedly rare in its scale and broad in its content, it had an unconventionally low response to its baseline survey of approximately 6%. This has prompted debates about the generalizability of its findings.^46,47,48,49^ This notwithstanding, HSE-SHS, an independent data set, had response rates in the normal range (64%-78%).^50^

## Conclusions

Risk factor–end point associations were not sensitive to the placement of mortality data on the death certificate. Using cause of death positioned anywhere on a death certificate may have the advantage of facilitating investigation of risk factors for the occurrence of rarer forms of death where, particularly in smaller cohort studies, there may be too few events to facilitate effect estimate computations using the underlying field alone.
